# Effect of Water Activity and Packaging Material on the Quality of Dehydrated Taro (*Colocasia esculenta* (L.) Schott) Slices during Accelerated Storage

**DOI:** 10.1155/2016/9860139

**Published:** 2016-11-09

**Authors:** A. R. Sloan, M. L. Dunn, L. K. Jefferies, O. A. Pike, Sarah E. Nielsen Barrows, F. M. Steele

**Affiliations:** Department of Nutrition, Dietetics and Food Science, Brigham Young University, S-221 ESC, Provo, UT 84602, USA

## Abstract

The quality of dehydrated taro slices in accelerated storage (45°C and 75% RH) was determined as a function of initial water activity (*a*_w_) and package type. Color, rehydration capacity, thiamin content, and *α*-tocopherol content were monitored during 34 weeks of storage in polyethylene and foil laminate packaging at initial storage *a*_w_ of 0.35 to 0.71. Initial *a*_w_ at or below 0.54 resulted in less browning and higher rehydration capacity, but not in significantly higher *α*-tocopherol retention. Foil laminate pouches resulted in a higher rehydration capacity and increased thiamin retention compared to polyethylene bags. Type of packaging had no effect on the color of the samples. Product stability was highest when stored in foil laminate pouches at 0.4*a*_w_. Sensory panels were held to determine the acceptability of rehydrated taro slices using samples representative of the taro used in the analytical tests. A hedonic test on rehydrated taro's acceptability was conducted in Fiji, with panelists rating the product an average of 7.2 ± 1.5 on a discrete 9-point scale. Using a modified Weibull analysis (with 50% probability of product failure), it was determined that the shelf life of dehydrated taro stored at 45°C was 38.3 weeks.

## 1. Practical Applications

Taro (*Colocasia esculenta* (L.) Schott) is a tuber grown in the tropics and subtropics. Though it contains high levels vitamins such as *α*-tocopherol and thiamine, it is a highly perishable root, resulting in high postharvest losses. In areas such as the Pacific Islands, where taro is a staple crop, this loss of nutrients is significant in the overall diet. Though some research has been done on the various ways taro can be preserved, little if any work has been reported on the effect of water activity (*a*_w_) and packaging type on dehydrated taro slices' quality and acceptability. The purpose of this research was to expand our understanding of the influence that packaging type and initial *a*_w_ have on stored, dehydrated taro's nutritional, physical, and sensory characteristics so as to increase shelf life, thereby reducing postharvest losses.

## 2. Introduction

Taro (*Colocasia esculenta* (L.) Schott) is a tuber crop grown throughout the tropics and subtropics. The total world production of taro in 2012 was about 9.97 million tons from an area of 1.32 million hectares, mainly in Africa, Asia, and Oceania [1]. Taro can be categorized into two main groups: the dasheen type, which has a large central corm (a thick, rounded stem base), with a few small side cormels, and the eddoe type, which has a small corm with large cormels. Raw taro is about three-quarters water with its main component being carbohydrate in the form of starch. In the Pacific Islands, taro (dasheen type) is an important staple food and plays a critical role in household, community, and national food security [2].

The major limitation to the use of taro is the scarcity of preservation methods, which leads to high postharvest losses. Taro corms can be stored at ambient tropical conditions for about 2 weeks before becoming unfit for human consumption due to postharvest rot [3–5]. This leads to heavy losses in developing countries where corms are stored at ambient temperatures, often at least 23.9°C [6]. In fact, Passam [5] reported average postharvest losses of taro of about 50% after six weeks of ambient storage. Research aimed at developing storable processed forms of taro would greatly contribute to minimizing postharvest losses of this staple crop [2].

Though some research has been done on the rehydratability and color characteristics of dehydrated taro slices during extended storage, little has been done on the effect of *a*_w_. A high temperature short time (HTST) pneumatic drying pretreatment prior to conventional hot air drying has been used to produce shelf-stable taro pieces with good rehydration and color characteristics [7]. In another study, Moy et al. [8] stored dehydrated taro slices in polyethylene (PE) bags at 21, 38, and 60°C for up to 6 months, finding that enzyme activity decreased and some browning occurred at 38 and 60°C. In another study, guava- and papaya-taro flakes stored at 38°C for 24 weeks maintained acceptable flavor; however, the ascorbic acid content and color of the samples were unstable during storage [9].

The impact of packaging on the color characteristics of dehydrated taro is unknown. However, in other food systems, it appears that packaging that maintains a low oxygen environment is most likely to preserve color. For example, in a study by Henríquez et al. [10], dried apple peel powder was stored in high-density polyethylene (HDPE) and metalized films of high barrier (MFHB), which is less permeable to oxygen than the HDPE. The MFHB packaging preserved most of the phenolic compounds, exhibited less moisture increase, and contributed to a longer shelf life. However, changes in packaging may not always have this effect on color. In a study on the color preservation of dehydrated green bell peppers by Sigge et al. [11], packaging type did not seem to affect color. Samples were stored in laminated pouches of different oxygen and moisture transmission rates; color changes due to storage temperature were significant, but changes due to packaging type were insignificant. Because of the variability of color changes due to packaging types in different food systems, it would be useful to specifically test the effects of packaging type on the color characteristics of taro.

Though color is certainly an important sensory characteristic and can also indicate the retention of some pigment nutrients, it is not always a good predictor of shelf life [12]. Therefore, shelf life should be studied separately.

An important aspect of shelf life is nutritional quality. Nutrient degradation during storage is typically a factor of storage temperature, pH, exposure to oxygen, porosity, light, and the presence of organic chemicals [13]. Taro is a good source of several vitamins, most notably *α*-tocopherol and thiamine, supplying 2.93 mg *α*-tocopherol and 0.107 mg thiamine for every 100 g of cooked taro [14]. This is 15% of the USDA recommended Daily Value for *α*-tocopherol and 7% of the recommended Daily Value for thiamine [15]. Due to the lack of previous research on the presence and stability of these vitamins in dried taro, a study of the stability of *α*-tocopherol and thiamine in dehydrated taro may be helpful.

Alpha-tocopherol is a fairly unstable, fat-soluble vitamin. It is degraded by storage and heat treatment, though it is stable at high temperatures if no oxygen is present [16]. Thiamine is a more stable molecule, but it can be heat sensitive, and interaction with SO_2_ can completely destroy thiamine [13]. Typically, the biggest concerns to thiamine degradation in food systems are time of heating, pH, and storage time [17].

In addition to nutritional aspects, some sensory aspects of dried taro products have also been investigated. In a consumer taste panel conducted in Fiji, 73% of panelists indicated they would consume dehydrated taro slices as a part of their regular diet [18]. Jayaraman et al. [7] stored HTST-dried taro pieces packed in paper-aluminum foil-polyethylene laminate pouches for 1 year under ambient conditions and found that the taro pieces retained both sensory acceptability and rehydration characteristics.

The Weibull distribution has been widely used in many shelf life studies. Hough and Garitta [19] name it the most statistically and experimentally sound methodology for shelf life estimations. It has been used on a variety of products, from coffee to baked products [20]. Because it is determined by the consumers' acceptance or rejection a product, it is most appropriate for studies in which the overall acceptability of a product is to be determined.

There exists a close relationship between *a*_w_ and many reactions involved in food stability such as microbial growth, enzymatic hydrolysis, oxidation, nonenzymatic browning, pigment loss, and nutrient loss [21]. Packaging with a poor moisture barrier allows the product to reabsorb moisture, which may lead to microbial growth, discoloration, and deterioration in flavor [22]. The aim of this project was to determine the effects of *a*_w_ and packaging material on physical, nutritional, and sensory properties of dehydrated taro slices during accelerated storage.

## 3. Materials and Methods

### 3.1. Experimental Design

An incomplete 4 × 2 × 4 factorial design was used with four levels of *a*_w_ (0.71, 0.54, 0.40, and 0.35), two levels of packaging material (foil laminate and polyethylene), and four levels of storage time (0, 13, 26, and 34 weeks). The amount of product required for a complete 4 × 2 × 4 factorial design exceeded the resources available due to limitations on the amount of product that could be dried during the available time. The design was incomplete in that investigating only one packaging material at *a*_w_ levels of 0.35 and 0.71 (as described below) allowed for the evaluation of a broader *a*_w_ range while still allowing a packaging material effect to be measured at *a*_w_ levels of 0.40 and 0.54.

### 3.2. Sample Preparation

Fresh corms of* Colocasia esculenta* (L.) Schott were obtained from a local market at Suva, Fiji. Corms were washed, peeled, and sliced with an electric food slicer (Model 213-A, Krups Gmbh, Solingen, Germany) into 0.5 cm thick slices for drying. Samples were randomly assigned to one of the four target *a*_w_ treatment levels (0.80, 0.70, 0.60, and 0.50). Drying was done using a forced-air tray dryer (Model FD-60, Nesco, Two Rivers, Wis., USA) at 57°C. During drying, the *a*_w_ of the samples was monitored by periodically testing the *a*_w_ of the largest taro slice in the batch using an Aqualab CX-2 water activity meter (Decagon Devices Inc., Pullman, Wash., USA) until the samples reached a high (0.80), intermediate (0.70), low (0.60), or very low (0.50) target *a*_w_ level. The samples were then placed in sealed PE bags for each *a*_w_ treatment level and transported under ambient conditions to the USA for evaluation.

In USA, samples were left in the PE bags and moisture was allowed to equilibrate at room temperature for 3-4 weeks. At equilibration, the *a*_w_ of three random samples from each bag was measured and the mean value for each bag was recorded. The resulting means (0.71, 0.54, 0.40, and 0.35) were designated as the actual *a*_w_ treatment levels. These treatment values were lower than the target levels because the samples were dried until the largest slice reached the target level to ensure that the majority of the taro slices were at the target *a*_w_ or below. Therefore, many of the smaller slices were below the target value at the time of drying, leading to lower final *a*_w_ values after equilibration.

After equilibration, samples at each of the 4 *a*_w_ levels were split into portions such that eight portions of approximately 276 g each could be randomly assigned to each of two types of packaging: PE bags (B01062WA, Nasco International, Fort Atkinson, Wis., USA) without oxygen absorbers and foil laminate (FL) pouches (Cal Pac 1500, Basaw Manufacturing, Inc., North Hollywood, Calif., USA) with oxygen absorbers (Ageless Z-Series, Mitsubishi Gas Chemical America Inc., New York, NY, USA). The FL pouches represent the most stability-enhancing packaging. The PE bags had a headspace oxygen level equal to atmospheric oxygen whereas FL pouches with oxygen absorbers provided a low (<0.01 ppm) headspace oxygen level, as measured using a 3500-Series Headspace Oxygen Analyzer (Illinois Instruments Inc., Johnsburg, Ill., USA). The PE bags (57 *µ*m thickness) were composed of a blend of low-density polyethylene and linear low-density polyethylene while the FL pouches (177 *µ*m thickness) were composed of layers of polyethylene, aluminum foil, and polyester. Both the PE bags and the FL pouches were sealed with a heat sealer (Model AIE-305 AI, American International Electric Inc., Whittier, Calif., USA). The samples with *a*_w_ of 0.35 were packaged only in the PE bags while the 0.71*a*_w_ samples were packaged only in FL pouches due to restrictions of product available. Each portion was packed separately, two portions for each of the four storage times. Packages for each treatment combination were stored in the dark in a controlled atmosphere chamber at an accelerated storage temperature of 45°C. The relative humidity (RH) of the storage chambers was set at 75%, simulating tropical humidity. Two portions of 138 g each were sampled from each treatment combination at 0, 13, 26, and 34 weeks for physical measurements and at 0 and 34 weeks for nutritional measurements.

At the conclusion of the 34 weeks of storage, random samples were taken from each treatment combination to measure any changes in *a*_w_ of the samples (Table 1). Only the treatments with initial *a*_w_ of 0.54 were compared when investigating headspace oxygen as a possible result of packaging differences because they had equilibrated to approximately the same *a*_w_ level (0.66).

### 3.3. Physical Analyses

*Water Activity.* Water activity was determined by the chilled mirror technique using an Aqualab CX-2 water activity meter (Decagon Devices Inc., Pullman, Wash., USA).

*Color.* CIE *L* ^*∗*^ (0 to 100, dark to light), *a* ^*∗*^ (±, green/red), and *b* ^*∗*^ (±, blue/yellow) color values were obtained by measuring reflectance with a Hunterlab Colorflex Spectrophotometer (Hunter Associates Laboratory, Reston, Va., USA) using a 64 mm glass sample cup. The mean values of triplicate measurements were reported.

*Rehydration.* Rehydration characteristics were determined on triplicate samples by immersing a taro slice (4–12 g) in 250 mL of distilled water at room temperature for 24 hours. The slice was drained for 2 min on a number 4 sieve (Fisher Scientific International Inc., Hampton, NH, USA) prior to weighing. Rehydration capacity was defined as the weight ratio between the water absorbed by the sample and the weight of the dehydrated sample [23].

### 3.4. Nutritional Analyses

Vitamin determinations were performed in a randomized order under subdued light. All chemical standards and enzymes were obtained from Sigma-Aldrich (St. Louis, MO, USA).

*Thiamin.* Thiamin was extracted in duplicate using the method of Ndaw and others [24]. Samples were ground using a coffee grinder (Model E160B, Hamilton Beach/Proctor-Silex, Inc., Glen Allen, VA, USA). Five grams of taro powder was placed in a 125 mL flask with 50 mL of 0.05 M sodium acetate that had been adjusted to pH 4.5. Ten mg papain, 10 mg acid phosphatase, and 10 mg *α*-amylase were added and mixed well. Samples were incubated at 37°C for 18 h. Thiamin was converted to thiochrome for quantification using an Agilent Model 1100 high performance liquid chromatograph (HPLC) (Agilent Technologies, Palo Alto, Calif., USA) and a Luna 5 *μ* C18 (2), 150 × 4.6 mm reverse phase column (Phenomenex, Inc., Torrence, Calif., USA). Separations were accomplished with the following HPLC conditions: mobile phase of methanol in 0.05 M sodium acetate (30 : 70, v/v); 23°C; 1 mL/min flow rate; 10 *μ*L injection volume; fluorescence detector, at an excitation wavelength of 366 nm and an emission wavelength of 435 nm. Data was quantified using an external standard.

*α*-*Tocopherol.* *α*-Tocopherol content was measured in triplicate using the method of Peterson and Qureshi [25] with modifications. Samples were ground using a coffee grinder (Model E160B, Hamilton Beach/Proctor-Silex, Inc., Glen Allen, VA, USA). Samples of taro powder weighing 1 g were extracted with 10 mL methanol, sonicated for 3 min, and centrifuged for 10 min. The supernatant was evaporated on a rotary evaporator (Model R-215, Buchi Labortechnik AG, Flawil, Switzerland) at 40°C and the residue was dissolved in hexane. Determinations were done using an Agilent Model 1100 HPLC (Agilent Technologies, Palo Alto, Calif., USA). The following HPLC conditions were used: mobile phase of hexane with 0.2% isopropanol; 23°C; 1 mL/min flow rate; 20 *μ*L injection volume; normal phase *μ* Prisol column (Waters Corp., Milford, Mass., USA), fluorescence detector, at an excitation wavelength of 295 nm and an emission wavelength of 330 nm. Data was quantified using an external standard.

### 3.5. Sensory Tests

Two sensory tests were performed. First, preliminary hedonic testing was performed on rehydrated taro slices in Fiji to determine general acceptability of the product. Second, a Weibull analysis of rehydrated taro stored in the US was conducted to determine shelf life of the product.

For the preliminary hedonic testing of rehydrated taro slices, eighty-six panelists (44 males, 25 of which were under 20 and 52 women, 25 of which were under 20) were recruited. These panelists were regular taro consumers who liked taro. Fresh taro of the same variety used in the study and of an approximate moisture content of 67% was purchased from the local market, peeled, and sliced in approximately 1 cm thick slices. The taro slices (about 8–12 g each) were dehydrated in a convection solar dryer, as described in Russon et al. [26], until they snapped when pressure was applied. Dried slices were then held for at least one week in FL pouches (Cal Pac 1500, Basaw Manufacturing, Inc., North Hollywood, Calif., USA) at ambient temperature, which is often at least 24°C, before sensory tests were conducted [6]. For the sensory test, the taro slices were rehydrated to as close to the original moisture content as possible (±3%) by boiling in 0.4% brine solution until the taro began to crack at the edges, fall apart, and turn gray, all of which are typical characteristics of taro doneness. Panelists seated at individual tables were presented a one-slice sample of taro to eat with their hands, as is typical in traditional consumption of taro. To analyze this product, panelists were given paper ballots to fill out. Panelists evaluated appearance, aroma, flavor, texture, and overall liking using a discrete 9-point hedonic scale where 9 = like extremely, 5 = neither like nor dislike, 1 = dislike extremely. The question about overall liking was placed at the end of the questionnaire to obtain a response that allowed time for panelists to consider all aspects of sensory quality, as has been done previously [27]. Acceptance was further determined by asking panelists if they would eat the sample as part of their regular diet and if they would eat it in an “emergency situation” in which there was a shortage of food.

Subsequent sensory analysis was conducted at Brigham Young University Sensory Laboratory (Provo, UT, USA) to determine shelf life. An abbreviated Weibull Hazard sensory analysis was conducted, modeled after that used by Cardelli and Labuza [28]. The end of shelf life was defined as the time at which 50% consumers found the product unacceptable.

Polynesians, including Fijians, who liked taro and consumed it occasionally were recruited as panelists from a database of campus and local communities. Both genders, across ages 18 to ≥60 years were represented. The university Institutional Review Board approved the study and panelists provided their informed consent. Panelists were monetarily compensated for their time.

Six panels were held after 0, 15, 26, 32.5, 39, and 42 weeks of storage. Beginning at week 26, tests were performed more frequently to ensure that the limit for shelf life could be determined accurately. The Weibull Hazard Method as described in Cardelli and Labuza [28] was used to determine the time of failure (end of shelf life) for the product and as a model for the method of conducting the panel. The first panel consisted of three panelists. The number of panelists was increased by *C* = 1 at each panel until 50% of the panelists deemed the samples unacceptable, as was done in other studies [19, 28, 29]. After that, the number of panelists was increased by *C* + *U*, with *U* = number of unacceptable responses for the previous test time, ending with a total of 15 panelists.

Dehydrated taro slices stored at 45°C in PE bags were used for the sensory panels conducted. They were representative of the taro used in the determination of *a*_w_, rehydration capacity, *α*-tocopherol content, and thiamine content. For each panel, slices of taro were soaked in distilled water at refrigerated temperatures for 15 hours and then boiled in a 0.4% brine solution until the taro began to crack at the edges, fall apart, and turn gray, indicating doneness. One slice of taro (8–12 g, 0.3–0.5 cm thick, and 5–8 cm diameter) was served to each panelist on a 15.24 cm diameter Styrofoam plate labeled with a three-digit blinding code. The sample was received through bread box-style compartments in isolated booths under normal 17 Watt fluorescent lighting. Panelists were instructed to use a bite of unsalted cracker and a sip of bottled water to cleanse the palate before tasting. Questions were presented one at a time on a computer screen and data was collected using Compusense®5 (version 4.6) software (Compusense Inc., Guelph, Ontario, Canada). Untrained panelists were asked to evaluate the samples as either “acceptable” or “unacceptable”, and to indicate whether or not they would consume the sample as part of their regular diet by answering either “yes” or “no.”

### 3.6. Statistical Analysis

Nutrient content data was analyzed using PROC MIXED of SAS (SAS Inst. Inc., Cary, NC, USA). Significant differences among treatment means were determined using Fisher's LSD for all pairwise comparisons with a significance level of 0.05.

Mean hedonic scores for the sensory analysis performed in Fiji were determined by averaging the 86 scores for the various aspects evaluated and calculating the standard deviations using a standard formula. Shelf life of taro slices was determined by a modified Weibull Hazard analysis, as presented in Cardelli and Labuza [28]. Hazard values were calculated as described in Gacula and Singh [29]. The log cumulative hazard was then plotted against time. The end of shelf life was determined to be the time for 50% probability that an untrained tester would grade the samples as being unacceptable. This probability corresponds to a cumulative hazard of 69.3.

## 4. Results and Discussion

### 4.1. Physical Quality

*Color.* The *L* ^*∗*^ (lightness) values ranged from 67.65 to 78.65 and showed no significant effects due to storage time, *a*_w_, or package type (Table 2). *a* ^*∗*^ (redness) and *b* ^*∗*^ (yellowness) values showed significant effects from storage time and *a*_w_. *a* ^*∗*^ and *b* ^*∗*^ values increased significantly with storage time (Tables 3 and 4). In the FL pouches, *a* ^*∗*^ and *b* ^*∗*^ values generally increased with increasing *a*_w_. This effect was not present in the PE bags, possibly because the samples equilibrated to the same *a*_w_ level or because of the greater headspace oxygen compared to the FL pouches. Nonenzymatic browning rate generally increases with increasing *a*_w_, reaches a maximum at *a*_w_ of 0.3–0.7, depending on the type of food, and decreases with a further increase in *a*_w_ [30]. Most enzymatic reactions are slowed at *a*_w_ < 0.8, which may explain the relatively light appearance of the dried taro, in as much as all *a*_w_ values studied were below this value. Water may accelerate both enzymatic and nonenzymatic browning by enhancing mobility of the reactants. On the other hand, an increase in water content may decrease browning rate by diluting the reactive components [31].

With respect to packaging material, the FL pouches did not result in less color change of the samples compared to the PE bags. However, if the FL packaging is utilized with lower initial *a*_w_, the dehydrated taro slices will likely experience less color change during storage.

**Figure 1:**
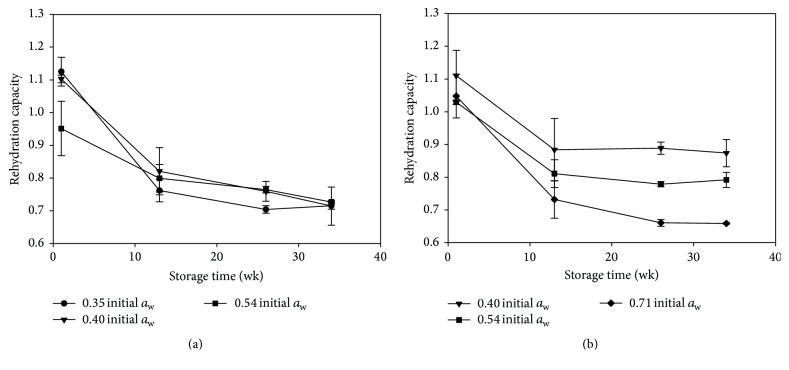
Dimensionless rehydration capacity of dehydrated taro slices dried to different *a*_w_ stored in (a) polyethylene bags and (b) foil laminate pouches at 45°C and 75% RH for 34 weeks (*n* = 3).

*Rehydration.* Storage time, *a*_w_, and packaging material had significant effects on rehydration capacity. Rehydration capacity decreased significantly with storage time (Figure 1). Most of the decrease in rehydration capacity occurred during the first thirteen weeks of storage. Sanjuán et al. [32, 33] observed similar results with dehydrated broccoli stored at 40°C. They noticed a sharp decrease in rehydration capacity occurring by 15 weeks of storage followed by a negligible decrease from that time forward. In the FL pouches, *a*_w_ was inversely correlated with rehydration capacity with lower *a*_w_ samples having higher rehydration capacities. The initial *a*_w_ of the samples in the PE bags had no effect on the rehydration capacity, again, likely due to the equilibrating of *a*_w_ levels to a relatively high level.

### 4.2. Nutritional Quality

*Thiamin.* Thiamin content is shown in Table 5. Results were adjusted to account for a recovery rate average of 85%. Storage time, *a*_w_, and packaging material had significant effects on thiamin content (*P* < 0.01). The initial thiamin content of the dried samples ranged within 2.03–2.54 *μ*g/g and did not show significant differences due to initial *a*_w_ levels prior to packaging. The thiamin content decreased significantly to 0.14–1.38 *μ*g/g [5.48–68.2% retention] after 34 weeks of storage. Retention of thiamin was highest in the FL pouches at 0.4*a*_w_. Both *a*_w_ and packaging material had significant effects on thiamin retention (*P* < 0.01). Degradation of thiamin generally increased with increasing *a*_w_. Dennison et al. [34] found similar results in a model system at 45°C; however, at temperatures ≤ 37°C thiamin was found to be quite stable regardless of storage temperature or *a*_w_. Thiamin retention was significantly higher in FL pouches. This could be due to the lower water vapor permeability which may have caused the samples to reabsorb moisture at a much slower rate than the samples in the PE bags. The higher thiamin retention could also have possibly been related to the exclusion of oxygen in the FL pouches.

*α*-*Tocopherol.α*-Tocopherol content is shown in Table 6. Results were adjusted to account for a recovery rate of 77% at 0 weeks storage and 84% at 34 weeks storage. Results are reported as *μ*g *α*-tocopherol per g of dry sample. Storage time, *a*_w_, and packaging material had significant effects on *α*-tocopherol content. The initial *α*-tocopherol content of the dried samples ranged within 1.59–9.97 *μ*g/g and was significantly lower at lower *a*_w_ levels, suggesting that it was susceptible to degradation during the prolonged drying required to obtain lower *a*_w_ levels. The *α*-tocopherol content generally decreased to values ranging within 1.30–7.59 *μ*g/g after 34 weeks of storage with the exception of the FL pouches at 0.40*a*_w_ which increased in *α*-tocopherol content. The results for that treatment combination are likely due to sampling variation within the treatment. The percent retention data was too variable to detect any differences in retention. *α*-Tocopherol content was generally higher in samples with higher initial *a*_w_. In contrast, Widicus et al. [35] found that *α*-tocopherol was less stable with increasing *a*_w_ and increasing molar ratio of oxygen in a dehydrated model food system at 37°C. If there was increased stability in taro at lower *a*_w_ levels it was secondary in importance to the effect of extended drying times on the initial content of *α*-tocopherol. *α*-Tocopherol content after storage was generally higher in the FL pouches. The exclusion of oxygen in the FL pouches may have slowed the rate of degradation of *α*-tocopherol.

### 4.3. Sensory Analysis

In the hedonic sensory test in Fiji, panelists rated the overall acceptability of the rehydrated taro at 7.2 ± 1.5 on a hedonic scale (Table 7). They also rated the appearance at 6.9 ± 1.9, the aroma at 7.0 ± 1.9, the flavor at 7.1 ± 1.8, and the texture at 7.0 ± 1.7. These results are congruent with “like moderately,” which is indicated by a score of 7.0 on the hedonic scale used. Of the 86 panelists, 73% indicated that they would accept this product into their regular diet, and 97% also said that they would use it in emergencies where there was a shortage of food. These results are consistent with results obtained by Rowe et al. [18], who conducted a consumer taste panel in Fiji, finding that 73% of panelists would consume dehydrated taro slices as part of their regular diet.

**Figure 2:**
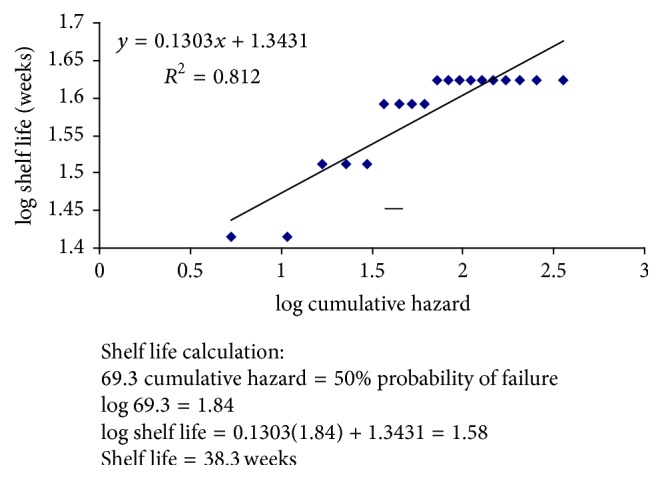
Weibull hazard plot for dehydrated taro slices stored at 45°C in PE.

For the subsequent sensory shelf life tests in the US, the ratings of each individual panelist are shown in Table 8. The product was deemed acceptable by 100% of the panelists for each panel until week 26 when some panelists began to deem it unacceptable. Weibull Hazard rankings were assigned for the failures in weeks 26 through 42, as shown in Table 9. Shelf life was determined to be 38.5 weeks as shown in Figure 2. In comparison, fresh taro's shelf life in tropical climates is typically only about 2 weeks [3–5]. The extension of shelf life of an acceptable taro product to 38.5 weeks is a substantial improvement.

As discussed earlier, PE's permeability to oxygen and moisture was associated with higher end *a*_w_ levels and greater headspace oxygen during storage. This higher *a*_w_ was associated with greater browning, lower rehydration capacity, and lower vitamin retention. This corresponds to Fennema's [21] observations which show the highest rate of browning, oxidation, and other nonenzymatic reactions in an *a*_w_ range of 0.6 to 0.8 of many food products. In the Weibull analysis conducted, increased browning and lower rehydration capacity could have had an impact on sensory quality, as they tend to indicate that a food is less fresh.

A sorption isotherm at 25°C, GAB model (data not shown), was run on the fresh taro to determine the effect of hysteresis on potential physical changes in the starch granules during dehydration and subsequent rehydration as described by Nurtama and Lin [36]; however, minimal hysteresis was observed which would support the high degree of acceptance in both the initial sensory test and the Weibull sensory data (Tables 7 and 8).

## 5. Conclusions

The quality of dehydrated taro slices in accelerated storage was investigated as a function of *a*_w_ and package type. Samples stored in low oxygen packaging (FL pouches) exhibited less *a*_w_ fluctuation during storage. Also, samples stored in FL pouches at lower *a*_w_ exhibited less browning and higher rehydration capacity. Thiamin retention was significantly higher in FL packaging and at lower *a*_w_. Alpha-tocopherol content was also higher in FL pouches, though it was likely susceptible to degradation during drying. It appears that packaging capable of maintaining a low *a*_w_, such as FL pouches, optimizes the physical quality of dehydrated taro slices during storage. In a hedonic test of dehydrated taro, panelists indicated that they liked product moderately, and in a Weibull analysis of dehydrated taro stored in PE bags, the shelf life was determined to be 38.5 weeks. If FL pouches were used instead of PE bags, the *a*_w_ may have remained lower, thus improving these two sensory impacting aspects. Thus, storing the dehydrated taro in FL pouches would likely increase the shelf life of this product even further. However, even this extension of shelf life through simple dehydration is not insignificant, especially as nutritional quality can be retained through the appropriate use of packaging and *a*_w_ level. Maintaining the nutritional and sensory attributes of taro can improve its functionality as an emergency or secondary significant food source in the Pacific Islands and elsewhere.

## Figures and Tables

**Table 1:** Initial and final *a*_w_ of dehydrated taro slices stored at 45°C and 75% RH for 34 weeks^a^.

Package type	Storage time (wk)	
	0	34
Polyethylene	0.35 ± 0.01	0.64 ± 0.06
Polyethylene	0.40 ± 0.004	0.65 ± 0.04
Polyethylene	0.54 ± 0.01	0.66 ± 0.03
Foil laminate	0.40 ± 0.004	0.56 ± 0.02
Foil laminate	0.54 ± 0.01	0.66 ± 0.02
Foil laminate	0.71 ± 0.01	0.73 ± 0.02

^a^Mean of two replicates ± standard deviation.

**Table 2:** Changes in *L* ^*∗*^ (0 to 100, dark to light) color values of dehydrated taro slices stored at different *a*_w_ levels and in different packaging materials^1^.

*a*_w_ level	Package type	Storage time (wk)			
		0	13	26	34
0.35	Polyethylene	74.32^abA^	79.22^aA^	68.23^bAB^	73.90^abA^
0.40	Polyethylene	70.61^aA^	72.57^aA^	72.07^aAB^	68.86^aA^
0.54	Polyethylene	70.90^aA^	72.11^aA^	76.68^aAB^	74.53^aA^
0.40	Foil laminate	78.73^aA^	70.51^aA^	73.13^aAB^	74.65^aA^
0.54	Foil laminate	73.83^aA^	70.41^aA^	78.65^aA^	73.26^aA^
0.71	Foil laminate	69.49^aA^	72.41^aA^	67.65^aB^	76.70^aA^

^1^Mean of two replicates.

^a-b^Means within a row with different small letters are significantly different (*P* < 0.05).

^A-B^Means within a column with different capital letters are significantly different (*P* < 0.05).

**Table 3:** Changes in *a* ^*∗*^ (±, green/red) color values of dehydrated taro slices stored at different *a*_w_ levels and in different packaging materials^1^.

*a*_w_ level	Package type	Storage time (wk)			
		0	13	26	34
0.35	Polyethylene	2.08^bA^	2.76^abA^	3.93^aAB^	3.46^abA^
0.40	Polyethylene	2.95^aA^	3.10^aA^	3.88^aAB^	4.08^aA^
0.54	Polyethylene	1.63^cA^	2.29^bcA^	3.25^abB^	3.95^aA^
0.40	Foil laminate	0.77^bB^	3.02^aA^	3.76^aAB^	3.15^aA^
0.54	Foil laminate	1.93^bA^	3.20^abA^	2.77^abB^	3.67^aA^
0.71	Foil laminate	2.71^bA^	3.68^abA^	5.17^aA^	4.57^aA^

^1^Mean of two replicates.

^a–c^Means within a row with different small letters are significantly different (*P* < 0.05).

^A-B^Means within a column with different capital letters are significantly different (*P* < 0.05).

**Table 4:** Changes in *b* ^*∗*^ (±, blue/yellow) color values of dehydrated taro slices stored at different *a*_w_ levels and in different packaging materials^1^.

*a*_w_ level	Package type	Storage time (wk)			
		0	13	26	34
0.35	Polyethylene	11.58^cA^	13.61^bcB^	17.09^aB^	15.64^abB^
0.40	Polyethylene	13.21^aA^	13.56^aB^	16.41^aB^	15.60^aB^
0.54	Polyethylene	11.70^bA^	13.72^abB^	15.20^aB^	16.51^aB^
0.40	Foil laminate	12.05^bA^	15.34^abAB^	17.35^aB^	16.82^aB^
0.54	Foil laminate	11.93^bA^	15.79^aAB^	16.75^aB^	18.49^aAB^
0.71	Foil laminate	12.59^cA^	17.72^bA^	21.52^aA^	21.01^abA^

^1^Mean of two replicates.

^a–c^Means within a row with different small letters are significantly different (*P* < 0.05).

^A-B^Means within a column with different capital letters are significantly different (*P* < 0.05).

**Table 5:** Thiamin content of dehydrated taro slices stored for 34 weeks at different *a*_w_ levels and in different packaging materials^1^.

*a*_w_ level	Package type	Initial content (*μ*g/g)	Final content (*μ*g/g)	Retention (%)
0.35	Polyethylene	2.51^aA^	0.45^bC^	18.01^C^
0.40	Polyethylene	2.54^aA^	0.14^bC^	5.48^D^
0.54	Polyethylene	2.47^aA^	0.17^bC^	6.82^D^
0.40	Foil laminate	2.03^aB^	1.38^bA^	68.20^A^
0.54	Foil laminate	2.54^aA^	0.95^bB^	37.46^B^
0.71	Foil laminate	2.16^aAB^	0.34^bC^	15.66^C^

^1^Mean of two replicates.

^a-b^Means within a row with different small letters are significantly different (*P* < 0.05).

^A–D^Means within a column with different capital letters are significantly different (*P* < 0.05).

**Table 6:** *α*-Tocopherol content of dehydrated taro slices stored for 34 weeks at different *a*_w_ and in different packaging materials^1^.

*a*_w_ level	Package type	Initial content (*μ*g/g)	Final content (*μ*g/g)	Retention (%)
0.35	Polyethylene	1.59^aC^	1.30^aC^	82.06^B^
0.40	Polyethylene	3.02^aC^	2.03^aC^	66.86^B^
0.54	Polyethylene	6.69^aB^	2.90^bBC^	44.64^B^
0.40	Foil laminate	2.79^bC^	4.45^aB^	160.57^A^
0.54	Foil laminate	9.97^aA^	7.59^bA^	77.59^B^
0.71	Foil laminate	8.83^aA^	6.64^bA^	75.23^B^

^1^Mean of three replicates.

^a-b^Means within a row with different small letters are significantly different (*P* < 0.05).

^A–C^Means within a column with different capital letters are significantly different (*P* < 0.05).

**Table 7:** Mean hedonic scores and percent acceptance from Fijian cultural acceptability sensory panel for dehydrated taro slices^a^.

Appearance	6.9 ± 1.9
Aroma	7.0 ± 1.9
Flavor	7.1 ± 1.8
Texture	7.0 ± 1.7
Overall acceptability	7.2 ± 1.5
Regular diet acceptance	73%
Emergency use acceptance	97%

^a^Mean of 86 scores ± standard deviation.

**Table 8:** Weibull sensory data for dehydrated taro slices stored at 45°C in PE bags.

Weeks	Acceptability														
0	+	+	+												
15	+	+	+	+											
26	−	+	+	+	−										
32.5	−	+	−	+	+	−									
39	−	+	−	+	+	+	+	+	−	−					
42	+	−	+	−	−	+	−	−	+	−	−	−	−	−	+

+: acceptable sample as assessed by an untrained panelist.

−: unacceptable sample as assessed by an untrained panelist.

**Table 9:** Weibull hazard ranking table for dehydrated taro slices stored at 45°C in PE bags.

Rank	Weeks	*H* value^a^	∑*H*
19	26	5.26	5.26
18	26	5.56	10.8
17	32.5	5.88	16.7
16	32.5	6.25	23
15	32.5	6.67	29.6
14	39	7.14	36.8
13	39	7.69	44.5
12	39	8.33	52.8
11	39	9.09	61.9
10	42	10.0	71.9
9	42	11.1	83
8	42	12.5	95.5
7	42	14.3	110
6	42	16.7	126
5	42	20.0	146
4	42	25.0	171
3	42	33.3	205
2	42	50.0	255
1	42	100.0	355

^a^ *H* = hazard value = 100/rank.
